# Decreased hospital readmissions after programmatic strengthening of an outpatient parenteral antimicrobial therapy (OPAT) program

**DOI:** 10.1017/ash.2022.330

**Published:** 2023-02-21

**Authors:** Gaurav Agnihotri, Alan E. Gross, Minji Seok, Cheng Yu Yen, Farah Khan, Laura M. Ebbitt, Cassandra Gay, Susan C. Bleasdale, Monica K. Sikka, Andrew B. Trotter

**Affiliations:** 1 College of Medicine, University of Illinois at Chicago, Chicago, Illinois; 2 Department of Pharmacy Practice, College of Pharmacy, University of Illinois at Chicago, Chicago, Illinois; 3 College of Pharmacy, University of Kentucky, Lexington, Kentucky; 4 Division of Infectious Disease, Department of Medicine, College of Medicine, University of Illinois at Chicago, Chicago, Illinois; 5 Division of Infectious Disease, Department of Medicine, Oregon Health & Science University, Portland, Oregon

## Abstract

**Objective:**

To determine whether a structured OPAT program supervised by an infectious disease physician and led by an OPAT nurse decreased hospital readmission rates and OPAT-related complications and whether it affected clinical cure. We also evaluated predictors of readmission while receiving OPAT.

**Patients:**

A convenience sample of 428 patients admitted to a tertiary-care hospital in Chicago, Illinois, with infections requiring intravenous antibiotic therapy after hospital discharge.

**Methods:**

In this retrospective, quasi-experimental study, we compared patients discharged on intravenous antimicrobials from an OPAT program before and after implementation of a structured ID physician and nurse-led OPAT program. The preintervention group consisted of patients discharged on OPAT managed by individual physicians without central program oversight or nurse care coordination. All-cause and OPAT-related readmissions were compared using the χ^2^ test. Factors associated with readmission for OPAT-related problems at a significance level of *P* < .10 in univariate analysis were eligible for testing in a forward, stepwise, multinomial, logistic regression to identify independent predictors of readmission.

**Results:**

In total, 428 patients were included in the study. Unplanned OPAT-related hospital readmissions decreased significantly after implementation of the structured OPAT program (17.8% vs 7%; *P* = .003). OPAT-related readmission reasons included infection recurrence or progression (53%), adverse drug reaction (26%), or line-associated issues (21%). Independent predictors of hospital readmission due to OPAT-related events included vancomycin administration and longer length of outpatient therapy. Clinical cure increased from 69.8% before the intervention to 94.9% after the intervention (*P* < .001).

**Conclusion:**

A structured ID physician and nurse-led OPAT program was associated with a decrease in OPAT-related readmissions and improved clinical cure.

Outpatient parenteral antimicrobial therapy (OPAT) is a treatment option for medically stable patients who require intravenous (IV) antimicrobial therapy in an outpatient setting. It has been utilized since the 1970s. OPAT permits decreased length of hospital stay in patients who require IV antimicrobial therapy, reduces cost to both the patient and the healthcare system, and reduces the risk of nosocomial infections.^1–4^ OPAT is not without limitations, however; unplanned 30-day hospital readmission rates as high as 26% have been reported.^5^ Moreover, intravascular catheters used for OPAT are associated with an increased risk for deep venous thrombosis and catheter-related infections.^1,6^

OPAT programs require the involvement of multiple providers including infectious disease (ID) physicians and often pharmacists and nurses, in addition to care coordination between multiple institutions including hospitals, outpatient clinics, home health agencies, infusion companies, rehabilitation facilities, and skilled nursing facilities.^1^ Due to the need for multiple providers and institutions to be involved in the care of patients on OPAT, communication and care coordination is critical to the feasibility, safety, and success of OPAT programs in successful treatment and in reducing hospital readmission rates for OPAT-related complications. According to the Infectious Diseases Society of America (IDSA) guidelines on OPAT, an effective OPAT team encompasses the physician, nurse, pharmacist, and the patient.^1^ Each individual in this collaborative team plays a unique role in the planning and delivery of high-quality care for patients discharged home on OPAT.^1^ The presence of a formal OPAT care team has been reported as one of the most important quality indicators in optimal OPAT care.^7^ However, not every institution with an OPAT program has dedicated personnel or a complete OPAT team as recommended by IDSA guidelines. In a 2018 survey of 672 ID physicians in the Emerging Infectious Network (EIN), only 36% had a structured OPAT program at their institution.^8^ An explanation for this may be inadequate hospital administrative support of OPAT programs (as reported by 59.8% of providers) or financial support (as reported by 64.6%).^8^ Lack of evidence demonstrating the value of dedicated staff for a successful OPAT program may explain why more resources are not allocated to OPAT programs. Studies that compare OPAT outcomes in OPAT systems, which utilize various team models, provide much needed evidence and justification that these models are effective.

The University of Illinois Hospital and Health Sciences System (UI Health) is a 463-bed tertiary-care academic hospital in Chicago. The evolution of the OPAT program at UI Health captures the spectrum of OPAT programs across the United States. Prior to hiring dedicated staff for the UI Health OPAT program, OPAT was managed by individual physicians who coordinated care individually without administrative support, dedicated care coordination, nor standardized management protocols.^9^ ID consultation was not required for OPAT; however, most OPAT patients (69%) described in a cohort from 2012 to 2013 at UI Health had an ID physician involved in their care at the time of OPAT initiation.^9^ Individual physician management was problematic for several reasons: nonstandardized structure of the OPAT program, significant time coordinating care, competing responsibilities of physicians, and an increasing number of OPAT patients in the program. In August 2017, a dedicated (1.0 full-time equivalent or FTE) registered nurse with nursing education, health utilization management, and discharge planning expertise was hired to provide centralized and standardized care coordination, to support patient communication, and to facilitate and document laboratory and pharmaceutical management for our OPAT program. In addition, an ID physician was designated to oversee the program (0.1 FTE) and a requirement was enacted that all OPAT orders and patient management would be directed by the OPAT team and an ID physician. An inpatient ID pharmacist was available to support the OPAT program as needed, but no pharmacist FTE support was dedicated. In this study, we examined risk factors for readmission of patients receiving OPAT and assessed readmission rates before and after the implementation of a structured OPAT program as a key programmatic indicator of OPAT functioning at a large, academic, tertiary-care hospital.

## Methods

### Study design

This retrospective quasi-experimental study included patients aged ≥18 years who received an ID consultation and were discharged from UI Health on OPAT. The preintervention group included patients discharged from UI Health with OPAT recommendations from the ID consultation service between January 1, 2012, and August 1, 2013. This cohort was a subgroup of patients from our previously published study.^9^ The postintervention group included patients discharged through the new structured OPAT program between October 1, 2017, and January 1, 2019. The structured OPAT program consisted of a full-time, dedicated nurse who provided care coordination and communication with home health agencies, infusion companies, patients, ID attending physicians and fellows, the ID clinic, and nursing and rehabilitation facilities. In addition, a dedicated ID physician served as the medical director by providing oversight for the program. This medical director developed laboratory monitoring protocols and management, and enhanced documentation standards. Although the medical director supervised the management of the entire program and oversight and support for the OPAT nurse, clinical decisions after hospital discharge were made by the supervising ID physician, often with an ID fellow, who cared for the patient while in the hospital and created the OPAT treatment plan. In most instances, the medical director and the supervising ID physician were not the same individual. The OPAT nurse utilized laboratory monitoring protocols for laboratory review, actions, and adjustment of dose and frequency, in coordination with the supervising ID physician. The primary end point of this study was unplanned, OPAT-related readmissions during receipt of OPAT. Readmissions deemed related to OPAT included treatment toxicities, complications of venous access devices (eg, infection, thrombosis, or dysfunction), and relapse of infection being treated. Secondary end points included all-cause readmission during OPAT and treatment outcome (successful versus failed treatment). Successful treatment was defined as completion of planned duration of IV antimicrobial therapy or a transition from IV antimicrobials to oral antimicrobials. Failed treatment was defined as clinical signs or symptoms of infection with or without readmission to UI Health Hospital due to infection progression or recurrence while on OPAT. Predictors of OPAT-related readmission were also evaluated. Initial review was performed by a study team member, and an ID physician verified categorization of whether a readmission was related or not related to OPAT. The University of Illinois at Chicago Institutional Review Board approved this research study.

### Participants

Patients included in this study were retrospectively identified through the following sources: billing codes, orders for outpatient IV antimicrobials, a registry of patients who had a peripherally inserted central catheter (PICC) placed while in the hospital, and a registry of patients enrolled in the structured OPAT program. Patients must have received their medication through a PICC line for a minimum of 2 days for treatment of an infection as an outpatient to be included. Patients were included if they were receiving IV antimicrobials after hospital discharge in any setting except for those who were receiving IV antimicrobials during scheduled hemodialysis sessions who were excluded. Each patient was followed for the entire duration of their IV antimicrobial therapy even if it was completed outside the enrollment period.

### Data collection and variables

The UI Health electronic medical record (EMR; Cerner, Kansas City, MO) was utilized to collect patient information and data was stored using REDCap (Research Electronic Data Capture). Data abstracted included patient general characteristics such as age, sex, comorbidities, insurance status, presence of assigned primary care physician. In addition, healthcare utilization measures were collected, including hospital length of stay, previous hospitalizations for any cause within past 12 months, intensive care unit (ICU) stay during hospitalization, primary clinical team upon discharge, presence of ID consultation during hospitalization. Additional data included ID diagnosis, microbiology cultures, treatment information (ie, specific antimicrobial used and drug class), total duration of antimicrobial therapy including number of inpatient days (time from IV antimicrobial initiation in hospital to discharge), and outpatient days (time from discharge to actual completion of planned duration IV antimicrobials, premature stop prior to planned end date, hospital readmission, or patient death). Further data were collected including frequency of dosing, location of OPAT administration (patient’s home, infusion center, skilled nursing facility, hospital clinic, or subacute rehabilitation center), and outpatient follow-up appointment including with which specialty. Outcome data included unplanned OPAT-related readmission during OPAT, all-cause readmission during OPAT, reason for readmission, infection cure, infection relapse, and OPAT discontinuation due to OPAT related complication. The Charlson comorbidity index was calculated for each patient using collected clinical information.^10^

### Statistical analysis

Comparisons of nominal data, such as patient demographics and readmission rates, were evaluated using the Fisher exact test or the χ^2^ test. Continuous data were evaluated using the Mann-Whitney *U* test. Predictors of readmission were also evaluated; factors associated with readmission at a significance level of *P* < .10 in univariate analysis were eligible for testing in a forward, stepwise, multinomial, logistic regression to identify independent predictors of readmission. Data analysis was conducted with SPSS version 25 software (IBM, Armonk, NY).

## Results

This study included EMR data for 428 patients: 73 from the preintervention period and 355 from the postintervention period. Table 1 presents the baseline characteristics of the pre- and postintervention populations. In both groups, the most common indication for OPAT was bone and joint infections. Beta-lactams and vancomycin were the most used antimicrobials. In both cohorts, most patients received their OPAT at home and had government-funded insurance. About half of both cohorts had a hospital admission in the 12 months preceding receipt of their first known episode of OPAT.

**Table 1.** OPAT Patient Demographics and Factors Before and After the Intervention

Patient Demographics	Before the Intervention (N = 73), No. (%)^a^	After the Intervention (N = 355), No. (%)^a^	*P* Value
Age, median y (IQR)	52 (47–67)	57 (43.5–60.5)	.023
Sex, male	45 (61.6)	184 (51.8)	.126
**Antimicrobial indications**			
Bone and joint infection	41 (56.2)	133 (37.5)	.003
CNS infection	13 (17.8)	34 (9.6)	.041
Skin/soft-tissue infection	6 (8.2)	29 (8.2)	.989
Genital/urinary tract infection	2 (2.7)	36 (10.1)	.043
Intra-abdominal infection	2 (2.7)	34 (9.6)	.055
Endocarditis	1 (1.4)	11 (3.1)	.700
Pneumonia	0	4 (1.1)	>.999
Other	8 (11)	74 (20.8)	.051
**OPAT administration location**			
Home	44 (60.3)	190 (53.5)	.291
Skilled nursing facility	22 (30.1)	57 (16.1)	.005
Subacute rehabilitation facility	7 (9.6)	105 (29.6)	<.001
Infusion center	0	1 (0.3)	>.999
**Comorbidities**			
Myocardial infarction	5 (6.8)	4 (1.1)	.009
Congestive heart failure	6 (8.2)	28 (7.9)	.924
Peripheral vascular disease	4 (5.5)	27 (7.6)	.523
Cerebrovascular disease	4 (5.5)	26 (7.3)	.574
Dementia	3 (4.1)	9 (2.5)	.439
Chronic pulmonary disease	17 (23.3)	42 (11.8)	.010
Connective tissue disease	5 (6.8)	3 (0.8)	.002
Peptic ulcer disease	0	3 (0.8)	>.999
Diabetes, without complications	12 (16.4)	52 (14.6)	.696
Diabetes, with organ damage	4 (5.5)	67 (18.9)	.005
Chronic kidney disease, moderate-to-severe	15 (20.5)	49 (13.8)	.141
Hemiplegia or paraplegia	7 (9.6)	14 (3.9)	.067
Leukemia	2 (2.7)	5 (1.4)	.341
Malignant lymphoma	2 (2.7)	5 (1.4)	.341
Solid tumor, nonmetastatic	5 (6.8)	34 (9.6)	.461
Solid tumor, metastatic	0	19 (5.4)	.055
Liver disease, mild	1 (1.4)	12 (3.4)	.706
Liver disease, moderate to severe	3 (4.1)	23 (6.5)	.595
Acquired immunodeficiency syndrome	5 (6.8)	7 (2.0)	.038
Charlson comorbidity index score, median (IQR)	2 (0.5–3.5)	3 (1.5–4.5)	<.001
Hospital admission in the previous 12 mo	36 (49.3)	182 (51.3)	.761
ICU admission during hospitalization	31 (42.5)	98 (27.6)	.012
**Insurance status**			
Government-funded insurance	47 (64.4)	259 (73.0)	.139
Private insurance	23 (31.5)	79 (22.3)	.091
No insurance	3 (4.1)	17 (4.8)	>.999
Assigned primary care provider	20 (27.4)	221 (62.3)	<.001
**Microbiology**			
Positive culture	48 (65.8)	307 (86.5)	<.001
ESBL	2 (2.7)	27 (7.6)	.198
KPC	1 (1.4)	2 (0.6)	.430
VRE	2 (2.7)	12 (3.4)	>.999
MRSA	14 (19.2)	46 (13.0)	.163
Any multidrug-resistant organism	18 (24.7)	84 (23.7)	.856
Gram-positive pathogen	39 (53.4)	217 (61.1)	.222
Gram-negative pathogen	11 (15.1)	120 (33.8)	.002
**Antimicrobial**			
β-lactam	41 (56.2%)	277 (78.0)	<.001
Vancomycin	39 (53.4%)	110 (31.0)	<.001
Fluoroquinolone	1 (1.4%)	3 (0.8)	.528
Aminoglycoside	0	2 (0.6)	>.999
Macrolide	0	3 (0.8)	>.999
Daptomycin	8 (11.0)	19 (5.4)	.107
Aztreonam	1 (1.4)	3 (0.8)	.528
Linezolid	1 (1.4)	1 (0.3)	.312
Clindamycin	2 (2.7)	0	.029
Colistin and polymyxin B	2 (2.7)	1 (0.3)	.077
Antifungal	1 (1.4)	12 (3.4)	.706
Antiviral	0	3 (0.8)	>.999
Other	14 (19.2)	14 (3.9)	<.001
Planned duration of OPAT, median d (IQR)	42 (32–52)	36 (22.5–49.5)	.002
In-house treatment duration, median d (IQR)	8 (4.5–11.5)	7 (4–10)	<.001
Outpatient treatment duration, median d (IQR)	30 (19–41)	25 (12–38)	.068

Note. OPAT, outpatient parenteral antimicrobial therapy; IQR, interquartile range; CNS, central nervous system ICU, intensive care unit; ESBL, extended-spectrum β-lactamase–producing bacteria; KPC, *Klebsiella pneumoniae* carbapenemase–producing bacteria; VRE, vancomycin-resistant *Enterococcus*; MRSA, methicillin-resistant *Staphylococcus aureus*.

a Units unless otherwise indicated.

After implementation of the structured OPAT program, the unplanned readmission rate due to OPAT-related complications significantly decreased from 17.8% (13 of 73) to 7.0% (25 of 355; *P* = .003). Reasons for readmissions due to OPAT-related complications included infection recurrence or progression (20 of 38, 53%), adverse drug reaction (10 of 38, 26%), or line-associated issues (8 of 38, 21%). The all-cause unplanned readmission rate decreased after the implementation of the structured program (67 of 355, 18.9%) compared to the preimplementation period (19 of 73, 26%); however, this difference was not statistically significant (*P* = .165).

Factors associated with hospital readmission due to OPAT-related problems in univariate analyses are listed in Supplementary Table 1. Independent predictors of hospital readmission due to OPAT-related problems are listed in Table 2. The multivariate regression model revealed that patients in the structured OPAT program as well as older patients were less likely to be readmitted due to OPAT-related reasons. Conversely, vancomycin and a longer duration of outpatient treatment were independently associated with an increased risk of OPAT-related readmission.

**Table 2.** Factors Independently Associated With Unplanned OPAT-Related Hospital Readmission During OPAT

Factors	Readmitted (N = 38)	Not Readmitted (N=390)	Multivariate Analysis Odds Ratio (95% CI)	*P* Value
Enrollment in strengthened OPAT program, no. (%)	25 (65.8)	330 (84.6)	0.449 (0.208–0.968)	.041
Vancomycin, no. (%)	22 (57.9)	127 (32.6)	2.448 (1.203–4.984)	.014
Outpatient treatment duration, median d (IQR)	36 (23.5–48.5)	25 (13–37)	1.009 (1.002–1.019)	.019
Age, median y (IQR)	51 (40.5–61.5)	57 (47.5–66.5)	0.969 (0.946–0.989)	.007

Note. OPAT, outpatient parenteral antimicrobial therapy; CI, confidence interval; IQR, interquartile range.

Clinical cure or treatment failure was documented for 385 (90%) of 428 patients. Among them, clinical cure occurred in 91.4%. Clinical cure increased from 69.8% before the intervention to 94.9% after the intervention (*P* < .001).

## Discussion

In this quasi-experimental study, the presence of our structured OPAT program was associated with lower unplanned OPAT-related hospital readmission rates and increased rates of clinical cure. ID involvement in OPAT has already been shown to lower readmission rates in various studies.^11–14^ In a study of privately insured patients aged <65 years, Shah et al^12^ reported that an ID-led OPAT significantly reduced 30-day hospital and emergency room readmission compared to no ID consultation. Although ID staff were involved with both the pre- and postintervention groups, we specifically examined the impact of developing a structured OPAT program that included the hiring of a dedicated nurse.

Having multidisciplinary teams involved in OPAT may improve OPAT outcomes, including minimization of readmissions and adverse events.^14–19^ For example, the implementation of a transition-of-care OPAT bundle in Bronx, New York, involving ID physicians, nurses, social workers, program administrators, data managers, and home-infusion liaisons was associated with a reduction of all-cause 30-day hospital readmissions in a retrospective study by Madaline et al.^20^ Although our OPAT program did not have an extensive multidisciplinary team, our intervention did involve the hiring of a dedicated OPAT nurse to the team whose skills were salient to the care coordination and communication functions required for OPAT. We hypothesized that having a dedicated OPAT nurse whose main roles include care coordination, laboratory follow-up and management, and patient education may allow the earlier detection and close follow-up of outpatient adverse events, thus potentially avoiding hospital readmission.

Beta-lactams and vancomycin were the 2 most-used OPAT drugs before and after the intervention. Patients who received vancomycin were more likely to be readmitted, which may in part be explained by the increased rates of adverse outcomes associated with vancomycin compared to other antimicrobials used in OPAT.^21–23^ A prospective study by Keller et al^24^ of 644 patients discharged from an OPAT program at an academic medical center found vancomycin receipt to be associated with serious adverse OPAT outcomes, including 30-day readmission. Similar to other institutions, we monitored vancomycin troughs instead of area under the curve (AUC) for OPAT patients.^19,24^ Future studies should revisit the independent risk of readmission and vancomycin when monitored by AUC because AUC-based dosing may improve therapeutic target attainments and is now recommended instead of troughs.^25,26^

In our study, longer OPAT duration was associated with a significant increased risk of unplanned readmission (median, 36 days) compared with a relatively shorter OPAT duration (median, 25 days). A strength of our study was that we were able to collect the completed duration of outpatient treatment, which may differ from the planned duration set at the time of hospital discharge. This finding reinforces the importance of regular monitoring for adverse events, especially as outpatient treatment length increases.

Similar to Keller et al^24^ we also found older age to be protective for readmission, supporting recommendations that elderly patients can be safely treated with OPAT.^27^ Several studies have found no association between age and readmission.^5,9,28,29^ We are uncertain why the younger age group (median age, 51 years) was more likely to be readmitted; we agree with Keller et al^24^ that younger patient populations may be more noncompliant with therapy. A survey of 65 adults discharged from a tertiary-care hospital on OPAT revealed that patient self-reported nonadherence to therapy was associated with younger age (median, 30 years) as well as lack of time and low income.^30^ In addition to considering patient age and the potential risks of noncompliance, complexity of dosing regimen, social support, and convenience should all be assessed when prescribing OPAT.^30^

We did not find significant associations between readmission and Charlson comorbidity index, socioeconomic factors (utilizing insurance as a proxy), aminoglycoside use, or the presence of multidrug-resistant organisms. Inconsistent conclusions have been drawn regarding the association between readmission and these risk factors.^5,9,28,29^

Our study had several limitations. The retrospective study design may have introduced recall and selection bias. Our data only can capture what is documented in the medical record, and adverse events may have occurred that were not recorded. Our patient population was discharged from an urban, tertiary-care, academic medical center and may not reflect patients in other settings. Moreover, we had a greater number of patients with bone and joint disease which may not be generalizable to other patient populations. Several differences in baseline characteristics of the 2 groups could have affected outcomes, including more bone and joint infections, less culture positivity and culture-directed therapy, and more vancomycin and less β-lactam therapy in the preintervention group compared to the postintervention group. To mitigate these differences, our multivariate analysis included all factors potentially predictive of readmission that were identified in the univariate analyses. However, we were unable to capture readmissions to other hospitals. We did not include analysis of subsequent readmissions after the first readmission to allow for better comparison with our preintervention cohort and to avoid confounding factors. We defined successful treatment as completion of IV therapy regardless of whether the patient stopped all antibiotics at that point or was transitioned to oral therapy. Some patients may have relapsed with infection while on oral therapy after completion of IV therapy. Finally, we did not assess the impact of our structured OPAT program on outpatient ID follow-up rates or the availability of monitoring laboratories while on OPAT. Multidisciplinary OPAT teams have been associated with increased ID clinic follow-up rates,^20^ and a follow-up OPAT clinic visit has been associated with a lower 30-day hospital readmission rate compared to no follow-up visit.^13^

The implementation of an ID physician and nurse-led OPAT program significantly reduced unplanned OPAT-related hospital readmission rates and improved clinical cure rates, underscoring the importance of multidisciplinary teams. Enrollment in our strengthened OPAT program and older age were protective factors from readmission, whereas vancomycin administration and a longer outpatient treatment duration were associated with an increased risk of readmission.

## Supplementary material

For supplementary material accompanying this paper visit http://doi.org/10.1017/ash.2022.330. click here to view supplementary material
