# Supplementary material for: Decreased hospital readmissions after programmatic strengthening of an outpatient parenteral antimicrobial therapy (OPAT) program
Source: Antimicrob Steward Healthc Epidemiol. 2023 Feb 21;3(1):e33. doi: 10.1017/ash.2022.330 (PMC9972539; doi:10.1017/ash.2022.330)
Supplement: Supplementary file 1 [file S2732494X22003308sup001.docx]

Supplemental Table 1. Factors associated with hospital readmission due to OPAT related problems in univariate analysis

| **Factors** | **Readmitted**  **N=38** | **Not Readmitted**  **N=390** | **Univariate Analysis**  **p-value** |
| --- | --- | --- | --- |
| Peripheral Vascular Disease | 0 | 31 (7.9%) | 0.096 |
| Diabetes mellitus with end organ damage | 2 (5.3%) | 69 (17.7%) | 0.049 |
| Primary care provider | 15 (39.5%) | 226 (57.9%) | 0.028 |
| Enrollment in strengthened OPAT program, n (%) | 25 (65.8%) | 330 (84.6%) | 0.006 |
| Vancomycin, n (%) | 22 (57.9%) | 127 (32.6%) | 0.002 |
| Outpatient treatment duration, days, median (IQR) | 36 (23.5-48.5) | 25 (13-37) | 0.002 |
| Age, years, median (IQR) | 51 (40.5-61.5) | 57 (47.5-66.5) | 0.003 |
| Charlson score, median (IQR) | 2 (0.5-3.5) | 3 (1.5-4.5) | 0.007 |
| Planned duration of OPAT, days, median (IQR) | 42 (29.5-54.5) | 36 (23-49) | 0.004 |
